# Adaptive Mesh Expansion Model (AMEM) for Liver Segmentation from CT Image

**DOI:** 10.1371/journal.pone.0118064

**Published:** 2015-03-13

**Authors:** Xuehu Wang, Jian Yang, Danni Ai, Yongchang Zheng, Songyuan Tang, Yongtian Wang

**Affiliations:** 1 Beijing Engineering Research Center of Mixed Reality and Advanced Display, School of Optics and Electronics, Beijing Institute of Technology, Beijing 100081, China; 2 Department of Liver Surgery, Peking Union Medical College Hospital, Chinese Academy of Medical Sciences and Peking Union Medical College, Beijing 100730, China; University of Nebraska Medical Center, UNITED STATES

## Abstract

This study proposes a novel adaptive mesh expansion model (AMEM) for liver segmentation from computed tomography images. The virtual deformable simplex model (DSM) is introduced to represent the mesh, in which the motion of each vertex can be easily manipulated. The balloon, edge, and gradient forces are combined with the binary image to construct the external force of the deformable model, which can rapidly drive the DSM to approach the target liver boundaries. Moreover, tangential and normal forces are combined with the gradient image to control the internal force, such that the DSM degree of smoothness can be precisely controlled. The triangular facet of the DSM is adaptively decomposed into smaller triangular components, which can significantly improve the segmentation accuracy of the irregularly sharp corners of the liver. The proposed method is evaluated on the basis of different criteria applied to 10 clinical data sets. Experiments demonstrate that the proposed AMEM algorithm is effective and robust and thus outperforms six other up-to-date algorithms. Moreover, AMEM can achieve a mean overlap error of 6.8% and a mean volume difference of 2.7%, whereas the average symmetric surface distance and the root mean square symmetric surface distance can reach 1.3 mm and 2.7 mm, respectively.

## Introduction

Computed tomography (CT) is one of the most commonly used techniques to produce cross-sectional images of the abdomen in clinical practice. CT is very useful in identifying numerous types of malignant liver tumors, such as metastatic tumors, hepatocellular carcinoma, and cholangiocarcinoma [1, 2]. This technique was proven powerful in detecting a large number of smaller lesions when integrated with arterial portography. CT has been reported to have an overall detection rate of 81% to 94% in identifying hepatic metastases [3, 4]. However, CT provides low intrinsic soft-tissue contrast, such that distinguishing the liver from adjacent organs, such as the gallbladder, stomach, and the heart, becomes difficult. Liver segmentation can identify the liver contour from the other tissues on the basis of intensity or shape distributions, which can be used for graft volume estimation, hepatic surgery planning, or liver metastasis monitoring.

Accurate liver segmentation in a CT image remains the most challenging task in the image-assisted diagnosis of hepatic diseases [5]. Over the past decades, numerical approaches have been proposed to identify liver boundaries in a CT image. Ling et al. [6] proposed a hierarchical framework to monitor the propagation of the liver boundary in a coarse-to-fine manner, and then a learning-based technique was used for boundary deformation and shape detection. Ruskó et al. [7] proposed an automatic liver segmentation method by combining multiple phases of a contrast-enhanced CT image. In this method, the anatomical and multi-phase information is incorporated into the pre- and post-processing functions, such that the segmentation accuracy of the region-growing method can be significantly improved. Oliveira et al. [8] developed a deformable model implemented through level sets to segment the liver from a CT image. The parameters are adjusted by using a supervised optimization procedure. In this study, nodules and vessels are also segmented through a region growing process, which has been utilized for the surgical planning of liver tumor. To improve the runtime efficiency of large-scale sparse optimization for the shape priors of the liver, Zhang et al. [9] introduced a singular vector decomposition method to train the shape dictionary to be more compact. Furthermore, an affinity propagation method is applied to partition the surface into small sub-regions. In this method, the scale of sparse optimization is dramatically decreased, thus accelerating the segmentation process. Another approach proposed by Ciecholewski et al. [10] first extracts the liver contour through a finite number of connected polylines, after which the other tissues outside the liver contour are split into fragmental polygons and eliminated from the image. In addition to the methods introduced above, numerous interesting techniques have been presented in the “MICCAI 2007 Grand Challenge” workshop. In this workshop, both the raw CT image and the ground truth of the liver and portal-venous segmentation results were available for open test, thus becoming the public standard for the quantitative evaluation of liver segmentation methods.

The distinguishing capability of CT value remains limited. Thus, deformable model-based methods have been acknowledged to be among the most effective approaches to differentiate a specific tissue from other tissues in the abdomen. Baghdadi et al. [11] modified the deformable algorithm by adding three components, namely, the balloon, collision, and tube mask forces, to the external force of the deformable model. In addition, this approach capably segments multiple objects from magnetic resonance imaging scans of embryo images. Montagnat et al. [12] introduced a simplex mesh model that was regularized through the predefined shape constraint, which was integrated with the iterative closest point registration framework for the segmentation of the target from volumetric medical images. The shape description includes numerous degrees of freedom, such that the deformable model is well-suited for the modeling of complex shapes. Furthermore, as the external force of the deformable model is constrained by geometric transformation, the segmentation scheme reduces the degrees of freedom and improves the robustness of segmentation. Soler et al. [13] constructed reference models by delineating typical tissues, such as bones, skin, and spleen, which are further utilized to constrain the deformation of the geometrical model to the liver contours. Based on the segmented liver, portal veins are labeled, and hepatic anatomical structures are identified according to the Couinaud classification principle. To achieve accurate liver segmentation, several studies integrated the statistical shape model defined by a training set of shapes for the determination of the external forces of the deformable model [14–21]. For such applications, the statistical shape model needs to be registered to the CT image for accurate pose or global shape estimation of the liver. Once the average shape is aligned with the calculated CT image, local deformations of sharp corners or grooved regions can be determined by template matching procedures.

The deformable model-based methods first construct a predefined geometrical shape, which then deforms in space on the basis of the combination forces of external and internal energies [22]. The external forces are usually defined by a gradient field or distance transform, which functions as the force that drives shape deformation toward the liver boundaries. Internal forces are employed to maintain the smoothness of geometric shape deformation. The geometrical shape of the deformable model is usually defined as a triangular mesh in space; hence, each triangle facet can be utilized to represent the local feature of the deformable object, and the deformation vector of the local feature is usually denoted by the normal vector of the triangle facet. The triangle facet usually connects with multiple neighboring vertices. Thus, local shape deformation is constrained by the neighboring connecting forces. The deformable simplex model (DSM) [23] is proposed to manipulate the nonparametric surface representation of the deformable model. The DSM algorithm first calculates the gravity center of all the triangular facets of the initialized model. These gravity centers are utilized as new vertices, and neighboring vertices are connected to form the new structure of the deformation model. The DSM representation of the surface guarantees that each vertex has only three connected neighboring vertices, which is advantageous for the construction of internal and external forces.

The DSM-based method integrates internal and external forces into the same optimization framework, which fully utilizes the intensity distribution and shape of the segmentation target. Hence, the method can achieve accurate liver segmentation without substantial human interaction and large statistical computation burden [11]. However, this deformable model-based method has four main limitations. First, the deformable model is susceptible to the initial model estimation and can thus be easily trapped into the local minimum. Second, the deformation of the model is constrained by an internal force, which prevents image features with large curvatures from being reached. Third, the deformable model can easily be self-intersected in some local regions with minimum external forces. Fourth, automatically determining the weighting coefficients of internal and external forces is difficult. Despite having been studied for several years, the deformable model-based method still required improvement.

Based on previous studies, a novel adaptive mesh expansion model (AMEM) is proposed for liver segmentation from a CT image. First, a small DSM is initialized inside the tissue of the liver, and the connecting relationships between the neighboring vertices are constructed to constrain the smoothness of the deformable model. Furthermore, the gradient images of the volume data are combined with the connecting forces to define the internal forces for manipulating the deformable model. Second, the balloon force is introduced to drive the expansion of the DSM, and the gradient and boundary images are combined to control model speed and extension. Third, the triangle facet of the DSM is adaptively decomposed into three smaller facets on the basis of the shape of the deformable model. This study has several contributions. First, a gradient image is used to constrain the tangential and normal forces, which can effectively preserve the smoothness of the deformation model. Second, the integration of the balloon force and the binarized image into the external force enables the precise manipulation of model deformation to drive the mesh model deform into irregular shapes. Such integration also prevents liver over-segmentation. Third, adaptive facet decomposition can adjust the size of the triangular facet according to the shape and extension of the target model, which can push the deformed mesh into narrow and long regions, such as the liver tips.

## Methods

**Fig 1:**
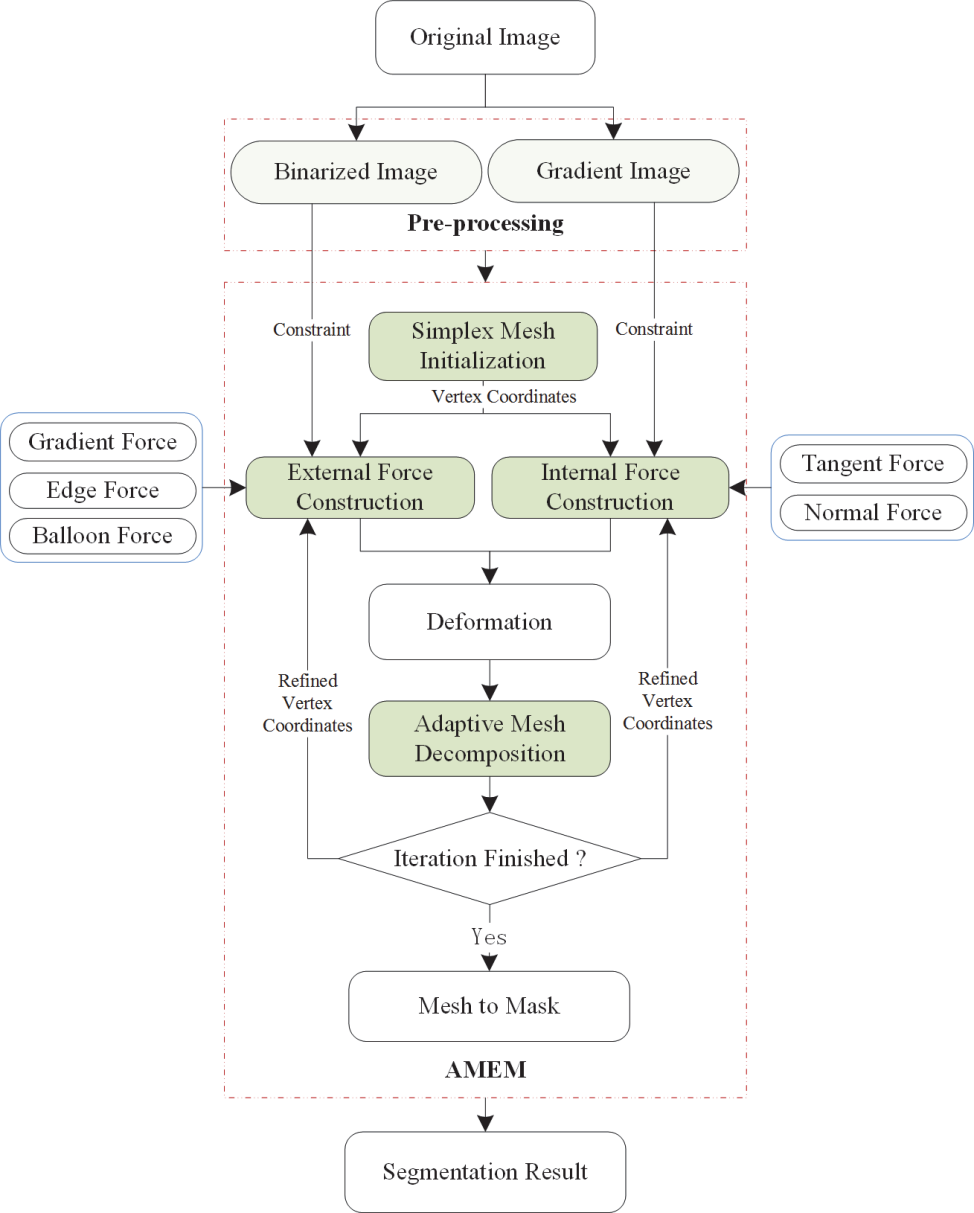
Flowchart of the proposed algorithm.

The proposed AMEM-based method consists of four main steps. The first step is image preprocessing. In this step, the abdominal CT image is smoothed through anisotropic filtering, and the binarized image containing fuzzy liver boundaries is obtained by calculating the gradient of the filtered image. The second step is the manual construction of DSM inside the liver tissue. The gradient, edge, and balloon forces are constructed and integrated as the external force, but tangential and normal forces are developed as the internal force. Hence, the DSM is deforming in space under the combination of internal and external forces. The third step is the adaptive decomposition of each triangle facet into three smaller triangle facets by adding a virtual vertex at the gravity center during deformable model expansion. Finally, the segmentation results are obtained at the end of the deformation. Fig. 1 shows the flowchart of the proposed algorithm.

### A. Image Pre-processing

The existence of noise may interfere with the segmentation result. Thus, the CT image is first smoothed through a nonlinear anisotropic diffusion filter to reduce noise and enhance liver boundaries. The density histogram is then obtained by assessing the intensity distribution of the whole image, after which the gradient image is obtained by calculating the first derivative of the smoothed image. Afterward, a binary image is obtained by stretching the whole image between two thresholds determined on the density histogram, which can effectively preserve the intensity of the liver and suppresses the intensity of other tissues. The obtained gradient image and the binary image are integrated into the internal and external forces to drive the deformation of the mesh model.

**Fig 2:**
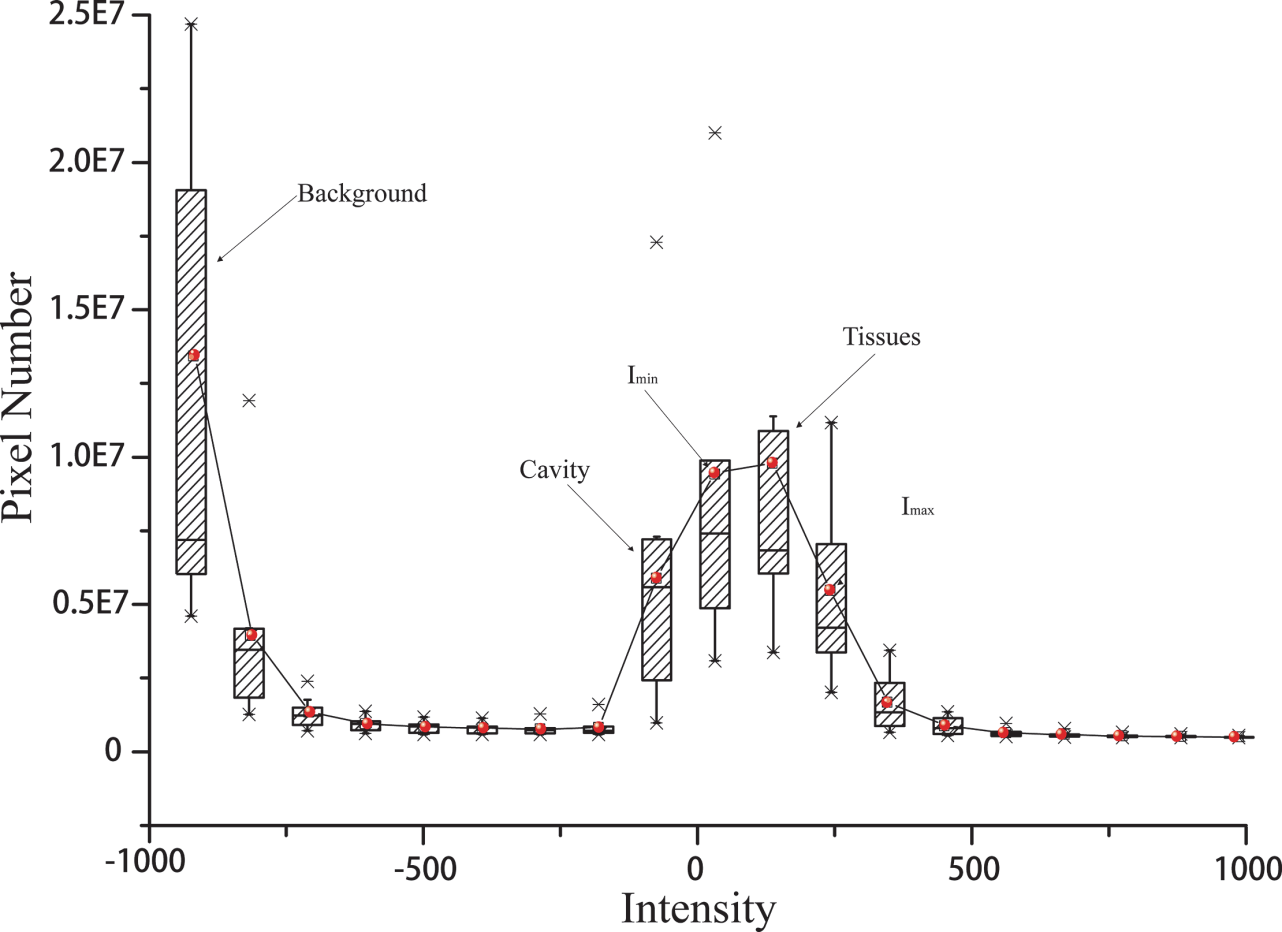
Histogram of thirty abdominal CT images.

To obtain the best thresholds for the segmentation of the liver, this study statistically analyzes the intensity distribution of the available abdominal CT images. Fig. 2 shows the histogram of the intensity distribution of thirty CT images, which illustrated the maximum, minimum, mean and the median values. From the figure, it can be seen that the background is mostly in the ranges of −1000 to −250. While the liver, heart and the kidney are in the intensity ranges of −250 to 500. Hence, we manually defined the maximum and minimum values, as illustrated as *I*_max_ and *I*_min_, for the fuzzy segmentation of the liver boundaries. Once the binarized image is obtained, the segmented boundary can be utilized as the constraint force for the mesh expansion model.

### B. The AMEM Algorithm

The internal forces of the deformable model generally comprise a stretching term and a bending term, which are utilized to preserve the smoothness of model deformation [24]. However, the degrees of smoothness and deformation are difficult to balance. If the force of smoothness is extremely large, the model cannot deform into the desired shape. However, if the deforming force dominates among the forces, the model may deform into several local maximums, such that the smoothness of the deformation model cannot be preserved [25]. To address this problem, the DSM is introduced to construct the internal and external forces. In this study, the shape deformation of the model is controlled by the vertices of the triangle facets instead of the normal vector of the facet.

**Fig 3:**
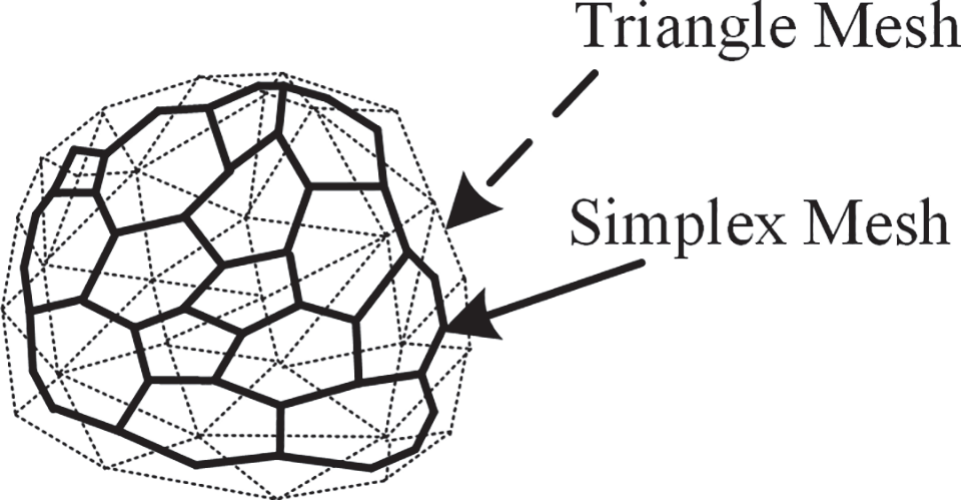
Relationship between simplex mesh and virtual triangle mesh.

**Initialization of AMEM.** The mesh of the proposed AMEM is composed of a vertex set {*p*_i_}, and each vertex is connected to three neighbors. Each vertex on the surface of AMEM is at the gravity center of a virtual triangle facet (dashed line), as shown in Fig. 3. The mesh of the AMEM is constructed as a dual triangle surface. The AMEM guarantees that more than three vertices are present for each facet of the surface. In addition, only three connected neighboring vertices are observed at the vertex on the surface.

For the constructed simplex AMEM, the motion of each vertex is determined by the combination of internal and external forces. According to the Newtonian law, the motion of vertex *P*_*i*_ can be defined as follows [26].

$$m\frac{\partial^{2}}{\partial t^{2}}P_{i}=-\gamma \frac{\partial }{\partial t}P_{i}+F_{\mathrm{internal}}+F_{external}$$ (1)

where *m* is the mass density of the surface, and *γ* is the damping factor. *F*_internal_ and *F*_*external*_ represent the internal and external forces, respectively. To represent the evolution of AMEM better under the combined forces, the time *t* is discretized, and we obtain the following regulation:

$$P_{i}^{t+1}=P_{i}^{t}+(1-\gamma )(P_{i}^{t}-P_{i}^{t-1})+\alpha_{i}F_{\mathrm{internal}}+\beta_{i}F_{external}$$ (2)

where *α*_*i*_ and *β*_*i*_ are the weighting coefficients of internal and external forces, respectively; and $P_{i}^{t}$ is the location of vertex *i* at the iteration *t*.

**Fig 4:**
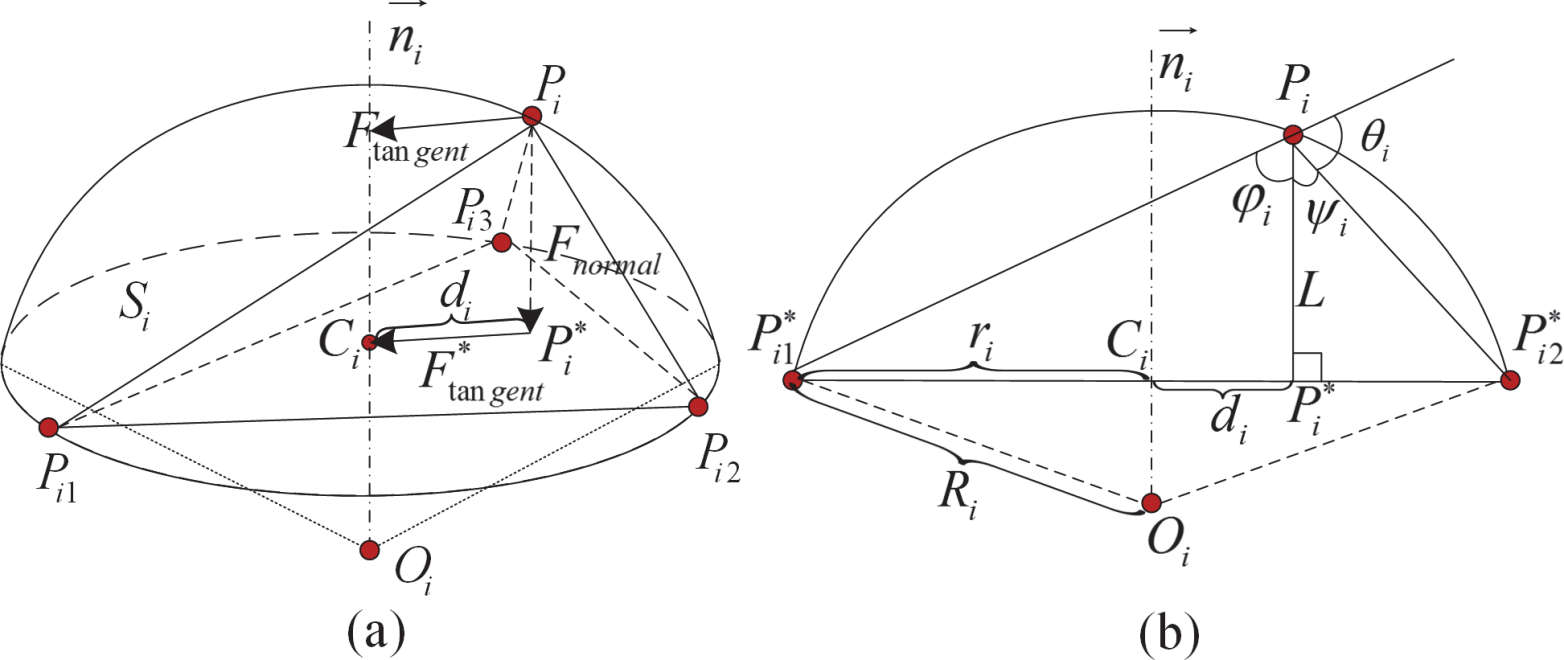
Force determination on the circumscribed sphere of the defined triangular mesh. (a) shows the construction of internal force for one triangle facet on the deformable model; (b) shows the cross-section between plane $\pi_{P_{i}P_{i}^{*}O_{i}}$ and the sphere *S*_*i*_.

**Internal Forces of AMEM.** The internal force of the proposed AMEM is constructed by combining the tangential and normal forces of the triangle facet on the deformable model, as shown in Fig. 4(A). Suppose that *P*_*i*1_, *P*_*i*2_ and *P*_*i*3_ are the three closest neighboring points of vertex *P*_*i*_ on the model surface. Let $\pi_{P_{i1},P_{i2},P_{i3}}$ represent the circle composed of points *P*_*i*1_, *P*_*i*2_, and *P*_*i*3_. Moreover, let $\vec{n_{i}}$ represent the normal vector of $\pi_{P_{i1},P_{i2},P_{i3}}$ and *S*_*i*_ represent the circumscribed sphere of a tetrahedron$V_{P_{i},P_{i1},P_{i2},P_{i3}}$. Let *O*_*i*_ represent the center of the sphere and *S*_*i*_. *C*_*i*_ represent the center of the circular section between plane $\pi_{P_{i1},P_{i2},P_{i3}}$ and sphere *S*_*i*_. Furthermore, let $P_{i}^{*}$ represent the projection of *P*_*i*_ on the plane$\pi_{P_{i1},P_{i2},P_{i3}}$, whereas *F*_*normal*_ and *F*_tan *gent*_ represent the normal and tangential forces at point *P*_*i*_, respectively. Fig. 4(B) shows the cross-section between plane $\pi_{P_{i}P_{i}^{*}O_{i}}$ and the sphere *S*_*i*_. Let *θ*_*i*_ represent the simplex angle and *R*_*i*_ represent the center of the sphere *S*_*i*_. Suppose that the internal force of the model is composed of normal and tangential forces, such that we have

$$F_{\mathrm{internal}}=\alpha_{\tan gent}F_{\tan gent}+\alpha_{normal}F_{normal}$$ (3)

Let vector $\vec{P_{i}P_{i}^{*}}$ represent the normal force *F*_*normal*_ at point *P*_*i*_. To calculate the normal force, the weighted coefficients *ω*_*i*1_, *ω*_*i*2_, and *ω*_*i*3_ are introduced to denote the distance relationship between *P*_*i*_ and its neighboring points *P*_*i*1_, *P*_*i*2_, and *P*_*i*3_. We thus obtain

$$P_{i}^{*}=\omega_{i1}P_{i1}+\omega_{i2}P_{i2}+\omega_{i3}P_{i3}$$ (4)

$$\omega_{i1}+\omega_{i2}+\omega_{i3}=1$$ (5)

Then, *P*_*i*_ can be denoted by the simplex angle *θ*_*i*_ as

$$P_{i}=\omega_{i1}P_{i1}+\omega_{i2}P_{i2}+\omega_{i3}P_{i3}+L(r_{i},d_{i},\theta_{i})\cdot \vec{n_{i}}$$ (6)

where *r*_*i*_ represents the radius of the circumcircle of$\pi_{P_{i1},P_{i2},P_{i3}}$, and *d*_*i*_ represents the distance between *C*_*i*_ and$P_{i}^{*}$. Moreover, the normal vector $\vec{n_{i}}$ at *P*_*i*_ can be defined as

$$\vec{n_{i}}=\frac{P_{i1}\times P_{i2}+P_{i2}\times P_{i3}+P_{i3}\times P_{i1}}{\left\|P_{i1}\times P_{i2}+P_{i2}\times P_{i3}+P_{i3}\times P_{i1}\right\|}$$ (7)

The relationship between *L*, *r*_*i*_, *d*_*i*_, and *θ*_*i*_ are illustrated in Fig. 4(B). Let *φ*_*i*_ represent the angle between $\vec{P_{i}P_{\text{i1}}^{*}}$ and$\vec{P_{i}P_{\text{i}}^{*}}$. Let *ψ*_*i*_ represent the angle between $\vec{P_{i}P_{\text{i}}^{*}}$ and$\vec{P_{i}P_{\text{i2}}^{*}}$. Thus, *φ*_*i*_ and *ψ*_*i*_ can be obtained by calculating the tangent of r_*i*_ and d_*i*_ with respect to *L* as follows: $\tan(\varphi_{i})=\frac{r_{i}-d_{i}}{L}$ and $\tan(\psi_{i})=\frac{r_{i}+d_{i}}{L}$. Given that *θ*_*i*+_ *φ*_*i*_ + *ψ*_*i*_ = π, we obtain the following Equation [27]:

L(ri,di,θi)=(ri2−di2)tan θiωri2+(ri2−di2)tan2θi+ri (8)

where *ω* is the constraint coefficient that determines the direction of the acting force. We then have

$$\omega =sign(\frac{\pi }{2}-\left|\theta_{i}\right|)$$ (9)

According to the plane geometry principle, *θ*_*i*_ can be calculated by the following three equations:

$$\sin\theta_{i}=\frac{r_{i}}{R_{i}}sign((\vec{P_{i1}P_{i}})\vec{\cdot n_{i}})$$ (10)

$$\cos\theta_{i}=\frac{\left\|\vec{O_{i}C_{i}}\right\|}{R_{i}}sign((\vec{O_{i}C_{i}})\vec{\cdot n_{i}})$$ (11)

$$\theta_{i}\in \left[-\pi ,+\pi \right]$$ (12)

Thus, the normal force at *P*_*i*_ can be calculated through the following equation:

$$F_{normal}=P_{i}-P_{i}^{*}=L(r_{i},d_{i},\theta_{i})\cdot \vec{n_{i}}$$ (13)

The liver is composed of soft tissue, which exhibits numerous sharp corners and a sag area. To segment the liver boundary accurately, this study introduces the theory of anisotropic diffusion to calculate internal forces. Normal force is used to constrain the curvature of the surface through the simplex angles *θ*_*i*_ and$\theta_{i}^{*}$. In this study, the normal force is determined by the distance between *P*_*i*_ and its maximum gradient point at the direction of $\vec{n_{i}}$, such that we have

$$\alpha_{normal}=\frac{1}{1+{\left|\nabla \left(G_{\sigma }\left(x,y,z\right)*I\left(x,y,z\right)\right)\right|}^{2}}$$ (14)

where *I*(*x*,*y*,*z*) is the intensity of liver at the sample point (*x*,*y*,*z*), ∇ is the gradient operator, and *G*_*σ*_(*x*,*y*,*z*) is the Gaussian kernel function with a standard deviation of *σ*.

In contrast to the normal force, the tangential force is designed to control the motion of the surface vertex with respect to its neighboring vertices. To calculate the tangential force of vertex *P*_*i*_, three weighting coefficients$\omega_{i1}^{*}$, $\omega_{i2}^{*}$, and $\omega_{i3}^{*}$ are introduced. These coefficients are determined by the distances between *C*_*i*_ and the three neighboring vertices. Thus, the tangential force can be defined as follows:

$$F_{\tan gent}=C_{i}-P_{i}^{*}=(\omega_{i1}^{*}-\omega_{i1})P_{i1}+(\omega_{i2}^{*}-\omega_{i2})P_{i2}+(\omega_{i3}^{*}-\omega_{i3})P_{i3}$$ (15)

Evidently, the tangential force is inversely proportional to the maximum gradient point in the normal direction of *P*_*i*_. The tangential force is smaller for the point closer to that with the maximum gradient. In the proposed method, the weighting coefficient of tangential force can be defined as follows:

$$\alpha_{\tan gent}=1-\frac{1}{1+{\left|\nabla \left(G_{\sigma }\left(x,y,z\right)*I\left(x,y,z\right)\right)\right|}^{2}}$$ (16)

Equation (3) can be rewritten as follows:

$$F_{\mathrm{internal}}=\left(1-\frac{1}{1+{\left|\nabla \left(G_{\sigma }\left(x,y,z\right)*I\left(x,y,z\right)\right)\right|}^{2}}\right)F_{\tan gent}+\left(\frac{1}{1+{\left|\nabla \left(G_{\sigma }\left(x,y,z\right)*I\left(x,y,z\right)\right)\right|}^{2}}\right)F_{normal}$$ (17)

External Force of AMEM. The external force is generally designed to drive surface model deformation [21, 28]. In this study, the external force is defined as the combination of gradient force *F*_*gradient*_ and boundary force *F*_*edge*_:

$$F_{external}=\beta_{i}^{grad}F_{gradient}+\beta_{i}^{edge}F_{edge}$$ (18)

Let *Max*_*grad*_ represent the image point with maximum gradient with respect to point P_*i*_ inside its circumcircle, such that the gradient force can be defined as follows:

$$F_{gradient}=(Max_{grad}-P_{i})\cdot \vec{n_{i}}$$ (19)

where $\beta_{i}^{grad}$ is the weighting coefficient with the value range of [0,1] and can be calculated as

$$\beta_{i}^{grad}=1-\exp\left(-\frac{\left|\nabla f\right|}{K}\right)$$ (20)

where *K* is a positive constant greater than zero, *f* is an edge map derived from the binarized image *I* ^*binary*^(*x*,*y*,*z*). Moreover, the edge map can be calculated as follows:

$$f={\left|\nabla \left(G_{\sigma }^{binary}\left(x,y,z\right)*I^{binary}\left(x,y,z\right)\right)\right|}^{2}$$ (21)

Hence, the edge force can be defined as the weighted distance between *P*_*i*_ and its neighboring voxel *Max*_*edge*_ with the largest intensity in the normal direction, which can be calculated as follows:

$$F_{edge}=Max_{edge}-P_{i}$$ (22)

where $\beta_{i}^{edge}$ is the stiffness of the edge force in the range of [0,1], and it can be defined as

$$\beta_{i}^{edge}=\exp\left(-\frac{\left|\nabla f\right|}{K}\right)$$ (23)

The initialized size and shape of the deformable model are important to the segmentation method-based deformable model [29]. To reduce the dependence of the initialization on the final deformation results, this study integrates the balloon force into the external force, which guarantees that the deformable model can rapidly expand to the boundary of the liver. The balloon force is defined as

$$F_{balloon}=q\cdot n_{i}$$ (24)

where n_*i*_ is the normal force at P_*i*_, and *q* is the weight coefficient. The external force of the deformable model can be defined as

$$F_{external}=B(I)\cdot (F_{gradient}+F_{edge}+F_{balloon})$$ (25)

where *B*(*I*) is Boolean type of weight coefficient. To avoid the overflow of the deformation model, we define *I*_min_ and *I*_max_ as the lower and upper thresholds for desired intensity values of the liver boundaries, respectively. Then, *B*(*I*) can be defined as

$$B\left(I\right)=\{\begin{array}{ll} 1, & \left(I_{\min}\leq I\leq I_{\max}\right)\\ 0, & \left(I_{\min}>I\;or\;I\leq I_{\max}\right) \end{array}$$ (26)

**Fig 5:**
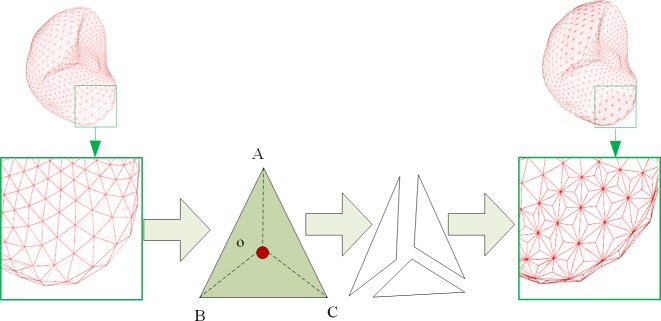
Adaptive triangular facet decomposition.

Adaptive Mesh Decomposition. During mesh expansion under the constraints of external and internal forces, the facet size of the mesh gradually increases for the model to reach the boundary of the liver. However, if the facet size is extremely large, the mesh cannot deform to several long and narrow regions. To segment the liver accurately, the adaptive mesh decomposition methods [30–32] reported by several studies are adapted. Most current methods drive the deformation of the triangular mesh by controlling the simplex angle *θ*_*i*_. Although such methods can effectively navigate the deforming degree of the mesh, the size of the triangular facet may become increasingly large with mesh expansion in space. Hence, the mesh can hardly deform into some long and narrow regions. The shape of the liver is irregular and has some sharp corners. Thus, previous methods cannot achieve high accuracy. In this study, a novel mesh decomposition method was proposed for the accurate segmentation of the long and narrow boundaries of the liver. The new vertex is adaptively inserted into the gravity center of the facet with size larger than a predefined threshold, which guarantees that the long and large facets will decomposed into smaller facets. At each step of the deformation, the triangular mesh is calculated from the simplex mesh, and then, the size of the enclosing area of the triangular facet is determined. If the area is larger than a predefined threshold, a vertex will be inserted at the center of gravity. At this point, the triangles will be broken into three smaller triangles, as shown in Fig. 5. The criterion is then iteratively applied on all the triangular meshes. The implementing flow of the algorithm can be found in Table 1. The proposed method certifies that a large and smoothed boundary comprises large facets, whereas long and narrow boundaries comprise small facets. This method is effectively balanced between calculation efficiency and accuracy.

**Table 1:** Pseudo code for local adaptive triangular facet decomposition.

Algorithm 2: Local Adaptive Method of AMEM
**Input:** Original triangular mesh {*O*_*i*_}
**Output:** Local adaptive triangular mesh{*L*_*i*_}
1$\begin{array}{l} 1\;:Definition:\\ \text{ Triangle mesh }\left\{O_{i}\right\}\text{, Number of triangle mesh }Number(O_{i}),\\ \text{ Barycenter of the triangle }Barycenter(O_{i}),\text{ Area of triangle}\\ \text{ mesh }Area(O_{i}),\text{ Threshold of Area Threshold, Number of }\\ \text{ iterations }T_{i}\\ 2:for\;i=0\text{ to }T_{i}\\ 3:\;\;\;\;\text{Deformation}\\ 4:\;\;\;\;for\;i=1\text{ to }Number(O_{i})\\ 5:\;\;\;\;\;\;\;\;\text{ Calculate the area of the triangle mesh }Area(O_{i})\\ 6:\;\;\;\;\;\;\;\;if\\ \;Area(O_{i})>Threshold\;then\\ 7:\;\;\;\;\;\;\;\;\;\;\text{Calculate the barycenter of the triangle}\\ \;Barycenter(O_{i})\\ 8:\;\;\;\;\;\;\;\;\;\;\text{Insert new vertex on }Barycenter(O_{i}),\;then\\ 9:\;\;\;\;\;\;\;\;\;\;\text{The triangle change into three smaller triangles}\\ \;\left\{O_{i}\right\}\to \left\{O_{i1},O_{i2},O_{i3}\right\}\\ 10:\;\;\;\;\;\;\;\;\;else\\ \text{ Deformation}\\ 11:\;\;\;\;\;\;\;\;\;end\;if\\ 12:\;\;\;end\;for\\ 13:\;\;\;\text{Refine the mesh of deformable model}\\ \;\left\{L_{i}\right\}\leftarrow \left\{O_{i1},O_{i2},O_{i3},\cdots \right\}\\ 14:end\;for\\ 15:return\;\left\{L_{i}\right\} \end{array}$:

## Results

To test the effectiveness of the proposed method, liver data sets obtained from the MICCAI 2007 grand challenge (http://www.sliver07.org/index.php) are used for the experiments. These data sets contain 20 training scans and 10 test data sets. All CT images are enhanced with contract agent and scanned in the central venous phase with various scanners. In addition, all data sets have been acquired in the transversal direction. The pixel spacing varies between 0.55 and 0.8 mm, whereas inter-slice distance varies from 1 to 3 mm. The algorithm was implemented in the C++ programming language under the Linux platform, and the experiments were carried out on a desktop computer with an i7–2600 processor and 16G memory.

### A. Visual assessment

**Fig 6:**
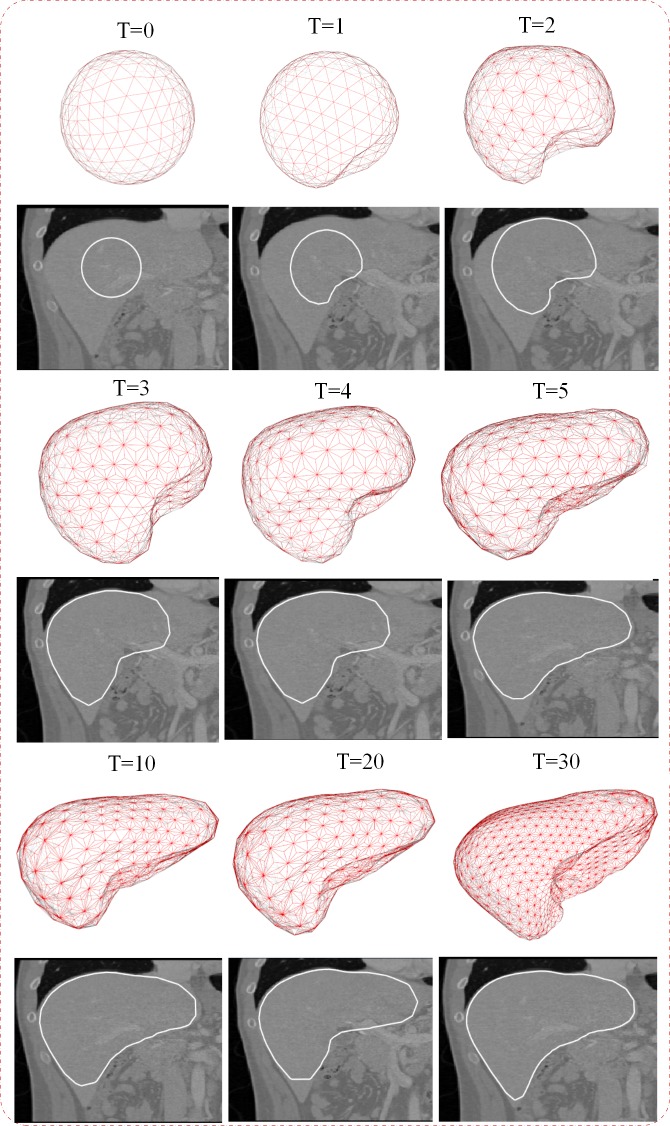
Nine intermediate deformation results of the proposed method. (T) shows the number of iterations.

Fig. 6 demonstrates nine steps of the intermediate calculation results of the proposed methods. The iteration number is denoted as T = 0 to T = 30, and the deformation model is superposed onto the original 3D volume data. From the Fig., it can be seen that the simplex mesh model gradually deforms from a spherical ball to the boundary of the liver. For the iteration steps 1 to 10, the degree of deformation is significantly fast, therefore, the mesh gradually deforms and finally reaches a steady state. Furthermore, triangular facet becomes finer with the increase of the iteration. Evidently, the mesh can deform into the sharp corners of the liver and obtain accurate segmentation.

**Fig 7:**
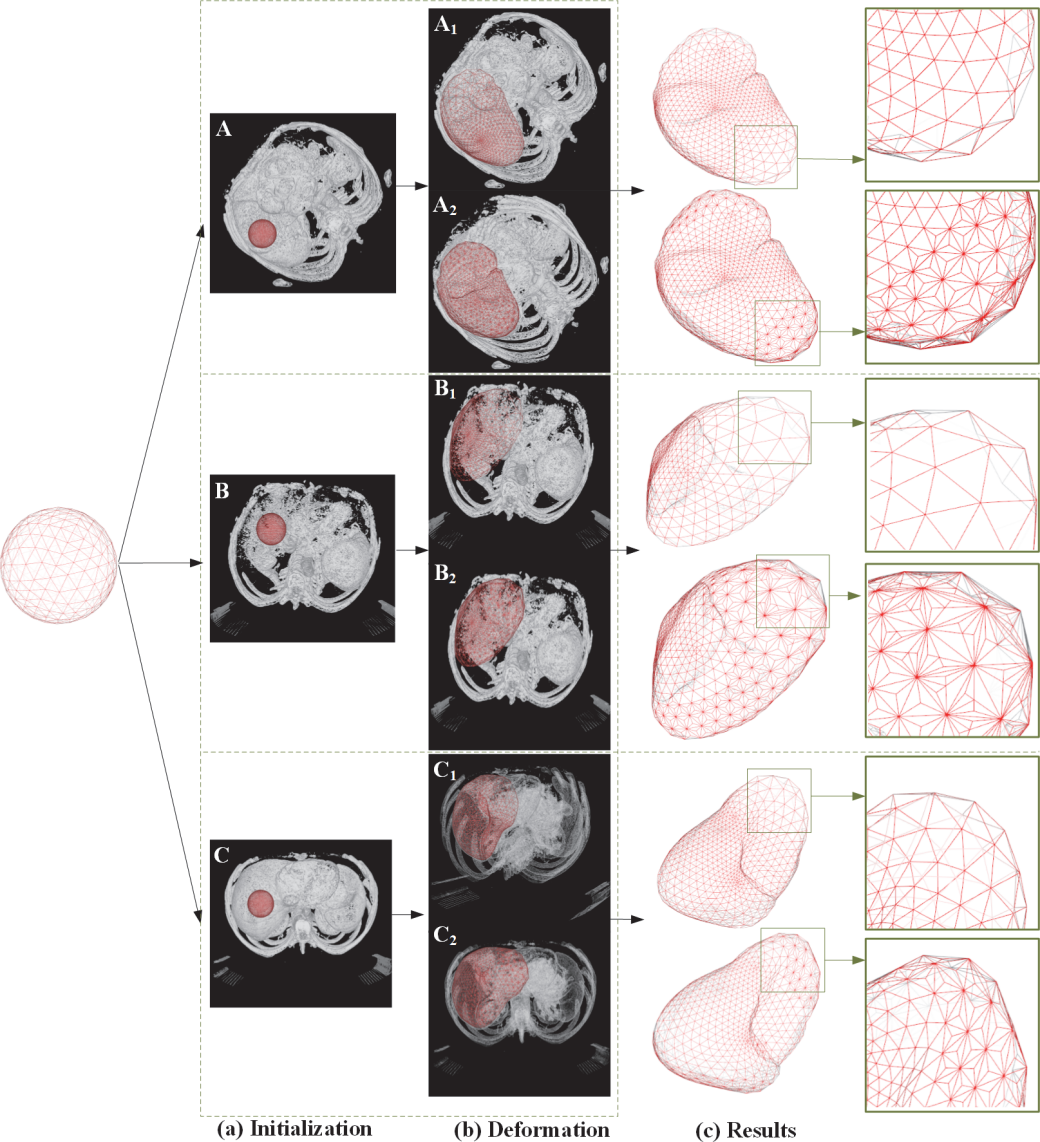
Segmentation results of the proposed method and the conventional DSM-based method. (A), (B) and (C) are three different data sets; (a) and (b) show the initialization and the finalized states of the deformation model; (c) shows the 3D meshes of the segmented liver.

Fig. 7 shows the segmentation results of the proposed method and conventional DSM-based method from the three abdominal volume data sets. The first column shows the initialized spherical mesh in space, and the second column shows the spherical mesh overplayed on the raw image data. The third column shows the intermediate segmented meshes of the liver overplayed on the raw images, and the fourth column shows the final segmented meshes of the liver in space. The fifth column shows the locally magnified views of the segmented meshes corresponding to the rectangular area of the fourth column. The proposed method accurately segmented the liver boundaries for the three provided volume data sets. The rows A_1_, B_1,_ and C_1_ show the segmentation results of conventional DSM-based method, and the rows A_2_, B_2,_ and C_2_ show the segmentation results of the proposed AMEM-based method. The segmentation results of the proposed method are finer than those of the conventional DSM-based method. Several local detailed structures can be accurately segmented from 3D volume data.

**Fig 8:**
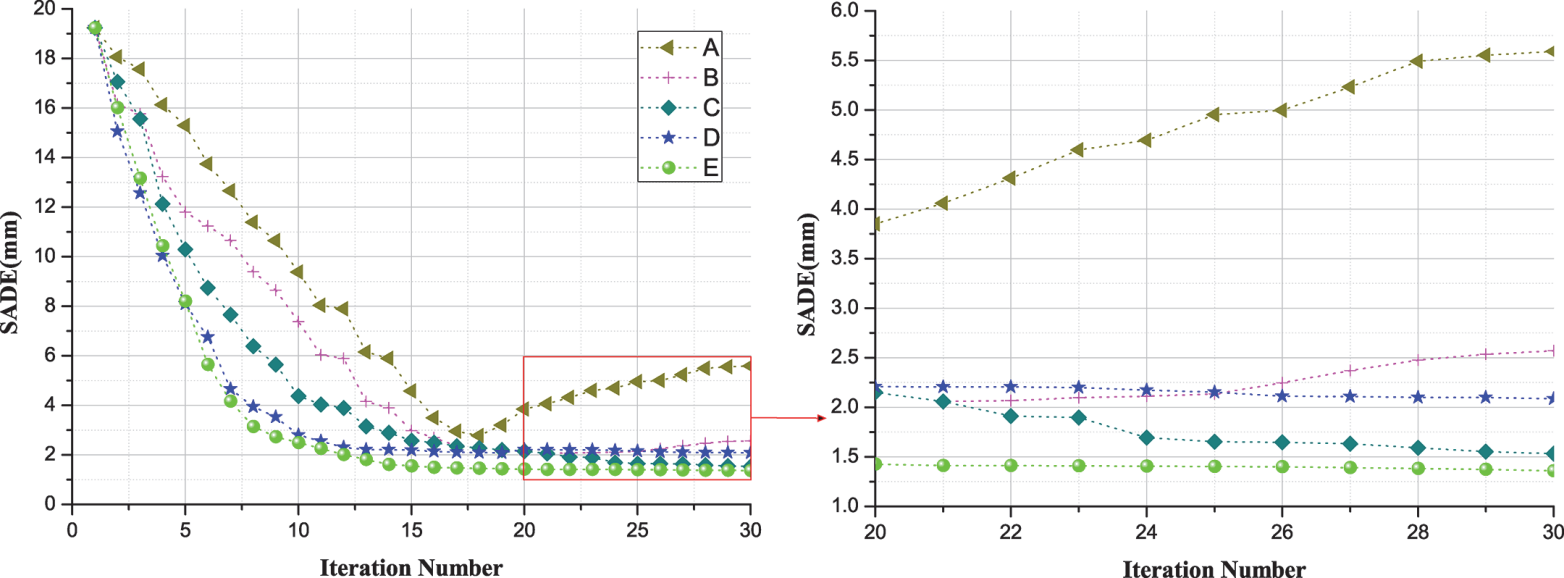
Comparison of the segmentation accuracies for different methods. (A) the conventional DSM; (B) DSM constraint by gradient image; (C) DSM constraint by the gradient and binary forces; (D) DSM constraint by gradient, binary, and balloon forces; (E) AMEM: DSM constraint by gradient, binary, balloon forces, and with adaptive triangular facet decomposition.

To validate the performance of the main procedures on the final segmentation results of the proposed AMEM, the segmentation results over each iteration step for the combined effects of the key individual processing techniques are compared and evaluated. In addition, the comparison models are designed as follows: (A) the conventional DSM; (B) DSM constraint by gradient force; (C) DSM constraint by the gradient and binary forces; (D) DSM constraint by gradient, binary, and balloon forces; and (E) AMEM: DSM constraint by gradient, binary, balloon forces, and with adaptive triangular facet decomposition. The segmentation error is quantified as the spacing average distance error (SADE) of all the vertices between the deformable model and the true value. Fig. 8 compares the average space errors at every iteration step for the above-mentioned models. The left figure shows the calculation errors at all the iteration steps, and the right figure shows the magnified view of the last 10 iterations. The segmentation errors gradually decreases with the iteration applied from the first to the eighteenth iterations in all six methods. Furthermore, the SADE values for (D) and (E) rapidly decrease from 19.3 mm to 2.49 mm, and 2.79 mm after ten iterations, which converge more rapidly than those of (A), (B), and (C). As the value of SADE for (A) gradually decreases from the first to eighteenth iteration, and then, the value rapidly increases from 2.77 mm to 5.59 mm from the thirtieth iteration. The DSM failure is possibly caused by the failure of the constraint of the external force. In addition, (C), (D), and (E) are substantially robust for liver segmentation, which yielded steady segmentation results for the final 15 iterations. Moreover, the final segmentation results of SADE for (C), (D), and (E) are 1.53 mm, 2.08 mm, and 1.50 mm, respectively. Evidently, with the introduction of the constraint conditions, the segmentation results are more accurate and the algorithms converge faster. Hence, it can be concluded that the proposed AMEM is considerably effective for the segmentation of the liver.

**Fig 9:**
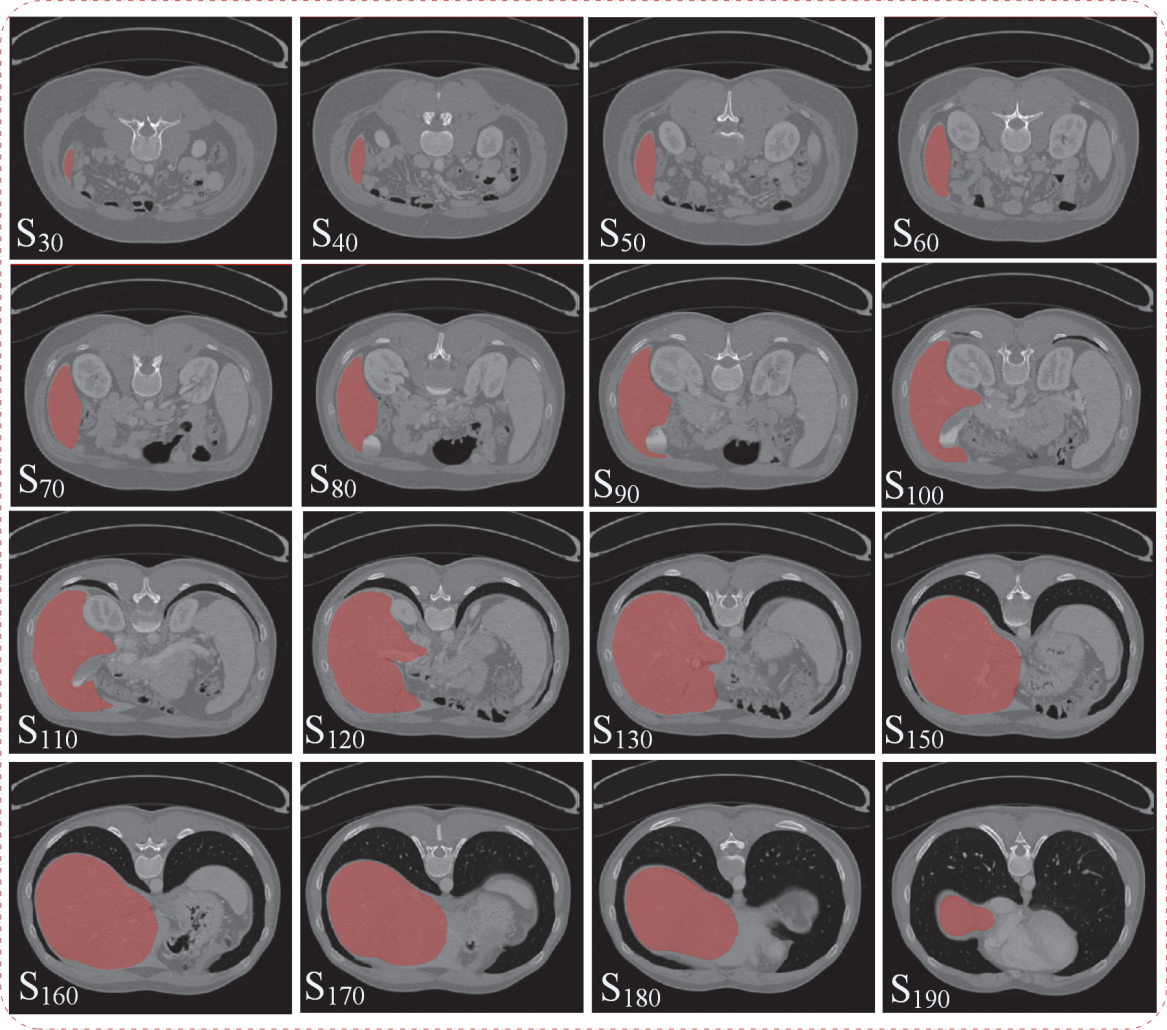
Final segmentation results of the proposed method over different image sections. (S) shows the number of the image section.

**Fig 10:**
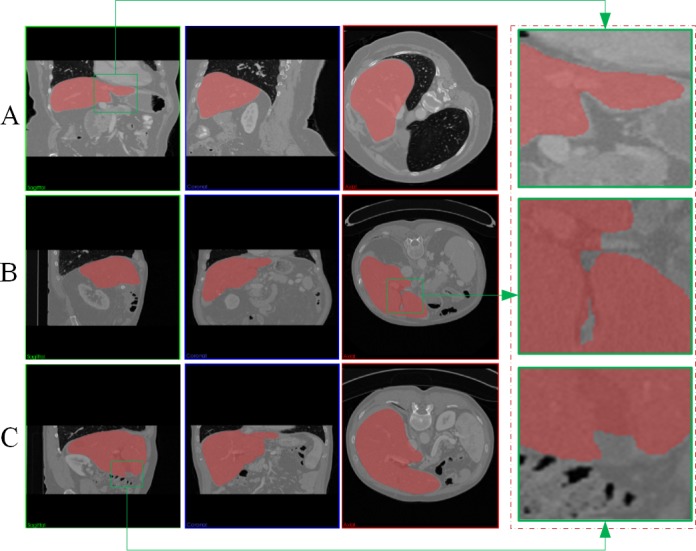
Segmentation results of the proposed method for three different CT data sets. (A), (B) and (C) demonstrate results of three different data sets.

Fig. 9 demonstrates the final segmentation results of the proposed AMEM-based method. In this fig., S_30_ to S_190_ represent the slice number of the 3D volume data in the transverse direction. The proposed AMEM-based method is significantly effective for the whole volume data of the liver. Although the intensity distribution of the liver in several regions is closer to the other tissues, the AMEM-based method is still capable of segmenting the liver from the background tissues. Fig. 10 shows the final segmentation results of the proposed method from three CT data sets. In this figure, the left three columns show the segmentation results in the transverse, sagittal, and coronal directions, and the fourth column shows the magnified views corresponding to the marked region in the left three columns. From the experiments, the sharp corner areas of the liver are precisely segmented by the proposed AMEM-based method.

**Fig 11:**
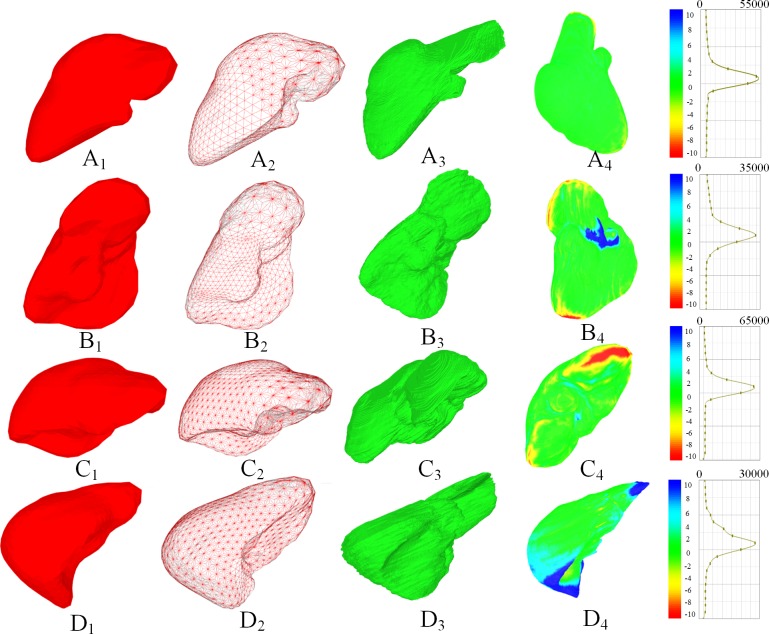
Segmentation results of four livers (A to D). (A_1_) to (D_1_) show the meshes of the liver; (A_2_) to (D_2_) show the wired grid of the liver; (A_3_) to (D_3_) show the ground truth of the liver surface; (A_4_) to (D_4_) show the color map of the segmentation error on the surface of the liver.

Fig. 11 shows four examples of the segmentation error distribution on the surface of the liver. The first to the fourth rows show the four different liver data sets. The first column shows the segmented mesh of the liver, and the second column shows the wired grid of the segmented mesh. The third column shows the ground truth segmentation results of the corresponding liver data, and the fourth column provides the color map of the segmentation error distribution on the surface of the liver. The fifth column provides the color bar and its corresponding density distributions. From the four groups of data sets, the segmentation results are steadily constant at the flat region of the liver, and most of the errors are distributed at the sharp corners or concave regions. In addition, the mean segmentation error is 1.6 mm, and the errors for 83.4% of the mesh vertices are less than 2.0 mm. evidently, the proposed method is significantly effective and robust for the segmentation of liver from CT images.

### B. Quantitative Comparisons

To further test the effectiveness of the proposed AMEM method, the method is compared with the other five commonly used segmentation methods over 10 groups of liver data. Suppose that *A* and *B* represent the segmented results and the ground truth for the evaluated data sets, then, the evaluation criteria can be defined as follows [33]: a) the volumetric overlap error between *A* and *B* (VOE); b) the relative volume difference between *A* and *B* (RVD); c) the average symmetric surface distance between *A* and *B* (ASSD); d) the root mean square symmetric surface distance between *A* and *B* (RMSSSD); and e) the maximum symmetrical surface distance between *A* and *B* (MSSD).

Table 2 lists the final segmentation results of the proposed AMEM from 10 different data sets. From the table, the proposed AMEM is significantly consistent for the liver segmentation. The calculated mean ratios of VOE, RVD, ASSD, RMSSSD, and MSSD are 6.8%, 2.7%, 1.3%, 2.7%, and 22.7%, respectively. Moreover, the obtained mean scores of VOE, RVD, ASSD, RMSSSD, and MSSD are 74, 85, 68, 62, and 70, respectively. For all tested data sets, the final segmentation score is 72. Table 3 compares the proposed method with the other seven commonly used methods, including the methods proposed by [7], [8], [18], [17], [20], [19], and [34]. The total scores of proposed AMEM method and above methods are 72, 71, 69, 67, 64, 62, 60, and 53. Evidently, this proposed method is the best among all the compared methods, and can obtain the highest segmentation score from the 10 groups of data sets. In addition, the proposed AMEM method achieves the highest MSSD value between the segmentation results and the ground truth. The proposed AMEM method also effectively demonstrates that the sharp corner of the liver is accurately segmented.

**Table 2:** Segmentation results of the proposed method over 10 groups of data sets.

**Data**	VOE		RVD		ASSD		RMSSSD		MSSD		**Total**
	[%]	Score	[%]	Score	[mm]	Score	[mm]	Score	[mm]	Score	Score
1	7.0	73	3.8	79	1.6	64	3.0	58	22.0	69	69
2	5.1	80	3.7	80	1.3	68	3.4	52	21.2	72	70
3	7.7	71	2.5	87	1.5	62	3.1	56	19.2	76	71
4	9.3	63	1.5	92	2.0	49	4.1	43	27.4	64	62
5	6.1	76	1.8	90	1.3	68	2.5	65	23.2	69	74
6	5.6	78	3.2	83	1.0	76	2.1	71	25.5	66	75
7	7.0	73	3.5	81	0.8	81	1.3	82	14.7	81	79
8	6.2	76	3.0	84	0.9	78	1.8	75	18.9	77	78
9	6.7	74	1.2	94	1.1	74	2.5	65	20.2	73	75
10	7.1	72	3.2	83	1.6	61	3.5	51	34.6	54	64
Mean	6.8±1.2	74±4.6	2.7±0.9	85±5.2	1.3±0.4	68±9.6	2.7±0.8	62±0.9	22.7±5.4	70±7.6	72±5.6

**Table 3:** Comparison of the segmentation accuracies of the proposed method and the other seven up-to-date methods.

**Methods**	VOE		RVD		ASSD		RMSSSD		MSSD		**Total**
	[%]	Score	[%]	Score	[mm]	Score	[mm]	Score	[mm]	Score	Score
Our	6.8±1.2	74±4.6	2.7±0.9	85±5.2	1.3±0.4	68±9.6	2.7±0.8	62±0.9	22.7±5.4	70±7.6	72±5.6
Ruskó [7]	8.2	-	1.7	-	1.3	-	2.6	-	23.3	-	71
Oliveira [8]	7.3	71	2.2	82	1.4	66	3.1	58	26.8	65	69
Heimann [18]	7.7±1.9	70	1.7±3.2	88	1.4±0.4	65	3.2±1.3	55	30.1±10.2	60	67
Saddi [17]	8.9±1.8	65	1.2±4.4	80	1.5±0.4	62	3.4±0.8	52	29.3±8.4	62	64
Chi [20]	9.1±2.8	65	2.6±6.3	73	1.7±0.6	58	3.3±1.2	54	30.8±9.2	60	62
Seghers [19]	10.4±2.5	58	6.8±2.3	64	1.8±0.4	55	3.2±1.1	56	25.2±10.1	67	60
Rikxoort [34]	12.5±1.8	51	1.8±4.2	80	2.4±0.3	40	4.4±1.5	40	32.4±13.7	57	53

## Conclusion

This study proposes a novel adaptive mesh expansion model for the 3D segmentation of livers from CT images. To precisely control the deformation of the mesh grid toward the boundary of the liver, this study introduces a simplex mesh model to depict the deformation model in 3D space. In this model, each vertex has only three connected edges, which makes the motion of the vertices easy to manipulate and drive the deformation of the mesh. To improve the calculation efficiency, this study integrates balloon, edge, and gradient forces into the external force of the deformable model. Therefore, the mesh model can rapidly deform in space to approach the target liver boundaries. In addition, this study introduces tangential and normal forces to control internal force, in which the smoothness of deformation model can be precisely controlled. In addition, the triangular facet of the deformable model is adaptively decomposed into smaller components according to the size and shape of the facet, which guarantees the segmentation accuracy of sharp corners of the liver.

The effectiveness of the proposed constraint conditions is evaluated by the spacing average distance error over every iteration step of the 10 groups of data sets. The experimental results demonstrate that the proposed constraint conditions, including gradient image, binary image, balloon force, and adaptive triangular facet decomposition, significantly improve the efficiency and convergence speed of the commonly used DSM algorithm. The proposed AMEM algorithm is also evaluated through the commonly used criteria of VOE, RVD, ASSD, RMSSSD, and MSSD over the 10 groups of randomly selected data sets. The experimental results show that our method is significantly effective and robust for liver segmentation with respect to most of the criteria and obtains the highest segmentation score of 72 among all evaluated methods. Currently, the average time consumed by the liver segmentation is about 2.5 minutes, which can still be significantly improved. Hence, future studies should be focused on graphics processing unit-based acceleration of the proposed method.
